# Myopericarditis After mRNA COVID-19 Vaccine in a Patient With Recent History of COVID-19

**DOI:** 10.7759/cureus.25264

**Published:** 2022-05-23

**Authors:** Eiman Elhouderi, Eman Elsawalhy, Mohamed Kabbani

**Affiliations:** 1 Internal Medicine, Beaumont Health, Dearborn, USA

**Keywords:** covid-19 infection, m-rna vaccine, pericarditis, myocarditis, covid-19 vaccine

## Abstract

Myopericarditis has been identified as a potential adverse event of several vaccines in the medical literature. Here we present a case of a 30-year-old male who had myopericarditis a week after receiving the second booster dose of the Pfizer-BioNTech coronavirus disease 2019 (COVID-19) vaccine. The patient's clinical course was not severe and had a full recovery after a week of treatment.

## Introduction

Myopericarditis is inflammation of the pericardial membrane that is associated with a mild degree of myocardial inflammation manifesting by elevation in cardiac enzymes (troponin). Recently, multiple cases of myopericarditis have been reported one week after receiving the second booster dose of mRNA coronavirus disease 2019 (COVID-19) vaccination as Pfizer-BioNTech and Moderna [1]. In the past, the same adverse event occurred after administration of vaccinations against other viruses such as smallpox virus. However, the exact mechanism is unclear [2]. Myopericarditis can be managed conservatively and most of the patients have excellent outcomes. Our patient had complete recovery after one week of management.

## Case presentation

**Figure 1:**
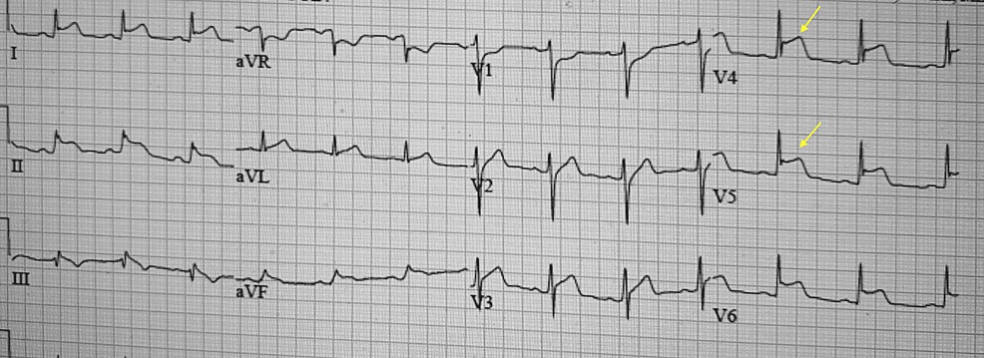
EKG showing ST-elevation in the inferior and anterolateral leads

A 30-year-old African American male with a history of COVID-19 infection two months ago presented to the hospital after one week of receiving a second dose of the Pfizer-BioNTech COVID-19 vaccine. He presented with midsternal chest pain, fever, chills, and shortness of breath. The vital signs on arrival included a blood pressure of 159/101, heart rate of 90 beats/minute, respiratory rate of 18 breaths/minute, and oxygen saturation of 98%. The physical exam was normal. The lab report was significant for elevated troponin of 2.9 ng/ml that continued trending up to 20.43 ng/ml and elevated inflammatory markers including C-reactive protein of 115.4 mg/l and erythrocyte sedimentation rate of 26 mm/hr. The COVID-19 polymerase chain reaction (PCR) test was negative. The chest X-ray was normal. EKG showed diffuse J-point elevation on inferolateral leads that progressed to 4-5 mm in the anterior, lateral, and inferior leads on the repeat EKG (Figure 1). Echocardiography showed normal left ventricular systolic function and no segmental wall motion abnormalities.

**Video 1:** Echocardiography, two chambers view.

**Video 2:** Echocardiography, long parasternal view

Given the up-trending troponin level and the progressive EKG changes, coronary angiography was done to rule out ischemic heart disease and revealed normal coronary arteries.

**Video 3:** Coronary angiography

**Video 4:** Coronary angiography

Given the recent history of vaccination and elevated inflammatory markers, the diagnosis was presumed to be myopericarditis related to the COVID-19 vaccine. The patient was managed with steroid and non-steroidal anti-inflammatory medications. He was then discharged in stable clinical condition. Follow-up in one week confirmed complete symptomatic recovery.

## Discussion

Myopericarditis is caused by different factors including infection, vaccines, drugs, autoimmune diseases, and radiation. Viral infection is the most common cause of myopericarditis. Myopericarditis is shown to be caused by different types of vaccines with the smallpox vaccine being the most common one [3,4]. Diagnosis of myopericarditis requires both fulfilling the criteria for pericarditis and the presence of finding of myocardial involvement [3,5]. Criteria for diagnosis of pericarditis include the presence of at least two of the following four criteria; typical chest pain, pericardial friction rub, ST-segment elevation or pericardial effusion. The clues for myocardial inflammation include focal or generalized ST elevation on EKG, the elevation of cardiac biomarkers or echocardiographic evidence of focal or global ventricular dysfunction with the exclusion of other causes for decreased ventricular function such as acute coronary syndrome. The diagnosis can be confirmed by the evidence of myocarditis on cardiac magnetic resonance imaging in the presence of elevated troponin or endomyocardial biopsy. In the appropriate clinical setting, the elevation of inflammatory markers such as C-reactive protein or erythrocyte sedimentation rate provides supportive evidence for the diagnosis of myopericarditis [5]. Myopericarditis can be treated as acute pericarditis with the use of non-steroidal anti-inflammatory drugs (NSAIDs), colchicine, or corticosteroid [3].

Interestingly, this case demonstrated that recent history (less than six months) of previous COVID-19 might be associated with an increased risk of COVID-19 vaccine myopericarditis. Fortunately, this case showed that elevated troponin and EKG abnormalities might not be associated with left ventricular dysfunction or poor outcome. 

COVID-19 infection is associated with myocarditis and pericarditis. A preprint from a population-based analysis showed that the incidence of myocarditis in young males is as high as 450 per million. However, observation studies from hospitalized patients showed a higher incidence than that [6]. The mechanism of COVID-19 induced myopericarditis is not known but is hypothesized that it is a result of immunological response to the virus rather than direct viral infection of cardiac myocyte [7,8].

As of March 3, 2022, the Center for Disease Control (CDC) has verified 1,337 reports of myocarditis or pericarditis associated with COVID-19 vaccines. The majority of cases have been reported after receiving the second dose of mRNA COVID-19 vaccines and in young males [9]. Myopericarditis has been also reported after the non-mRNA COVID-19 vaccine such as the AstraZeneca vaccine [10]. A case of a 30-year-old male who developed myopericarditis after the second dose of the Pfizer vaccine has been reported. The patient was tested negative for COVID-19 at the time of the presentation and he had very high Anti-spike IgG of more than 40.000 AU/ml [11]. A case series illustrated four cases of myocarditis that occurred 2-5 days after the second dose of mRNA COVID-19 vaccine have been published [12]. Both the case report and case series didn’t report if the patients have a history of prior COVID-19 infection. A literature review showed only one case report of myopericarditis after the first dose of the Pfizer vaccine in a patient with a confirmed recent prior history of COVID-19 [13].

Studies showed that self-reported previous COVID-19 is associated with 2.1 times higher risk of COVID-19 vaccine adverse events [14]. Moreover, the COVID-19 symptom study app illustrated that systemic side effects after the second dose of the Pfizer vaccine were higher in people with prior history of COVID-19 (38%) as compared to people with no history of prior COVID-19 (20%) [15]. In addition, studies showed that previous COVID-19 infection is associated with an increase in the peak level of Anti-spike IgG antibody after COVID-19 vaccines [16]. Since COVID-19 infection can be asymptomatic, especially in young people [17], it is difficult to confirm if patients who developed COVID-19 myopericarditis have prior COVID-19 infection or not. Our case report is the first case report to shed light on the possible association between recent prior COVID-19 infection and myopericarditis after the COVID-19 vaccine. Further studies are warranted to explore this association. 

## Conclusions

In summary, this case report showed that myopericarditis can be associated with the COVID-19 vaccine, particularly the mRNA vaccine. This adverse event occurs most commonly in young males. Previous COVID-19 infection might be associated with the increased risk of COVID-19-vaccine-related myopericarditis and further study is warranted to explore this association. This condition can be managed supportively and has a favorable outcome. Given the well-established COVID-19 infection mortality and morbidity, this potential rare adverse event should not lessen our confidence in the vaccine.
